# Supplementary material for: Health outcome priorities of people with multiple long-term conditions using the outcome prioritisation tool in the UK: A survey study and feasibility assessment
Source: PLoS One. 2024 Dec 31;19(12):e0301740. doi: 10.1371/journal.pone.0301740 (PMC11687667; doi:10.1371/journal.pone.0301740)
Supplement: S1 File — (DOCX) [file pone.0301740.s001.docx]

**SUPPLEMENTARY MATERIAL**

**Health outcome priorities of people with multiple long-term conditions using the outcome prioritisation tool in the UK: a survey study and feasibility assessment**

**Authors**

Sathanapally H, Chudasama YV, Zaccardi F, Rizzi A, Seidu S, Khunti K

Table of Contents

[Figure S1. Flow chart of survey respondents 2](#_Toc175301862)

[Table S1. Characteristics of survey respondents by the breakdown of categories 3](#_Toc175301863)

[Table S2. Prevalence of self-reported long-term physical or mental health conditions 4](#_Toc175301864)

[Table S3. Current first-choice health outcome priority by respondent sociodemographic factors and clusters of conditions 5](#_Toc175301865)

[Table S4. Association between patient’s first-choice health priority and sociodemographic factors and clusters of long-term conditions, unadjusted analyses 6](#_Toc175301866)

[Table S5. Association between patient’s first-choice health priority and sociodemographic factors and clusters of long-term conditions, adjusted for age and gender 7](#_Toc175301867)

[Table S6. Association between patient’s first-choice health priority and sociodemographic factors and clusters of long-term conditions, fully adjusted 8](#_Toc175301868)

[Table S7. Before COVID-19 first-choice health outcome priority by respondent characteristics, N (%) row totals 9](#_Toc175301869)

[Figure S2. Health outcome priority score before COVID-19 and current 10](#_Toc175301870)

[Figure S3. Comparing the health outcome priority score before COVID-19 and current 11](#_Toc175301871)

[Table S8. The health outcome prioritisation tool was easy to complete by patient’s sociodemographic factors and clusters of long-term conditions, N (%) 12](#_Toc175301872)

[Table S9. The health outcome prioritisation tool was relevant to my healthcare by patient’s sociodemographic factors and clusters of long-term conditions, N (%) 13](#_Toc175301873)

[Table S10. The health outcome prioritisation tool will be useful in communicating what my priorities are to my doctor by patient’s sociodemographic factors and clusters of long-term conditions, N (%) 14](#_Toc175301874)

# Figure S1. Flow chart of survey respondents

**19 GP practices across the East Midlands, UK**

**identified MLTC patients and sent the survey**

2,456 patients with MLTC completed survey

Excluded

Age <45 years 2

**No**

**Current OPT**

Missing an outcome 405

Same rankings 868

**Before COVID-19 OPT**

Missing an outcome 70

Same rankings 227

**N=1,570 (64%)**

**Yes**

Correctly answered the OPT current and before COVID-19

**N= 884 (36%)**

MLTC=multiple long-term conditions; OPT= outcome priority tool.

**N=2,454**

Descriptives analysis

OPT score

Feasibility test

**Applied the trade-off principle for OPT**

# Table S1. Characteristics of survey respondents by the breakdown of categories

| **Characteristics** | **Total** | **Applied trade-off principle to HOPT** | |
| --- | --- | --- | --- |
|  |  | **No** | **Yes** |
| **Total** | 2,454 | 1,570 (64.0) | 884 (36.0) |
| **Ethnicity** |  |  |  |
| White | 2,251 (91.7) | 1,430 (91.1) | 821 (92.9) |
| South Asian | 71 (2.9) | 46 (2.9) | 25 (2.8) |
| Black | 34 (1.4) | 22 (1.4) | 12 (1.4) |
| Mixed/ Other | 32 (1.3) | 22 (1.4) | 10 (1.1) |
| Missing | 66 (2.7) | 50 (3.2) | 16 (1.8) |
| **Education** |  |  |  |
| None | 445 (18.1) | 304 (19.4) | 141 (16.0) |
| GCSE or equivalent | 818 (33.3) | 533 (34.0) | 285 (32.2) |
| A Levels or equivalent | 347 (14.1) | 208 (13.3) | 139 (15.7) |
| Undergraduate degree or equivalent | 345 (14.1) | 207 (13.2) | 138 (15.6) |
| Postgraduate degree or equivalent | 270 (11.0) | 169 (10.8) | 101 (11.4) |
| Other (student, volunteer) | 178 (7.3) | 116 (7.4) | 62 (7.0) |
| Missing | 51 (2.1) | 33 (2.1) | 18 (2.0) |
| **Employment status** |  |  |  |
| Employed | 766 (31.2) | 515 (32.8) | 251 (28.4) |
| Self-employed | 121 (4.9) | 75 (4.8) | 46 (5.2) |
| Unemployed | 196 (8.0) | 127 (8.1) | 69 (7.8) |
| Retired | 1,143 (46.6) | 698 (44.5) | 445 (50.3) |
| Other | 172 (7.0) | 113 (7.2) | 59 (6.7) |
| Missing | 56 (2.3) | 42 (2.7) | 14 (1.6) |

Shown are the number of subjects (%).

# Table S2. Prevalence of self-reported long-term physical or mental health conditions

| **Self-reported conditions** | **Total**  N=2,454 | **Applied trade-off principle to HOPT** | |
| --- | --- | --- | --- |
|  |  | **No**  N=1,570 | **Yes**  N=884 |
| High blood pressure | 1,171 (47.7) | 747 (47.6) | 424 (48.0) |
| Arthritis | 890 (36.3) | 565 (36.0) | 325 (36.8) |
| Chronic pain | 703 (28.6) | 441 (28.1) | 262 (29.6) |
| Depression | 662 (27.0) | 421 (26.8) | 241 (27.3) |
| Anxiety | 659 (26.9) | 429 (27.3) | 230 (26.0) |
| Diabetes | 639 (26.0) | 410 (26.1) | 229 (25.9) |
| Other conditions* | 614 (25.0) | 384 (24.5) | 230 (26.0) |
| Asthma | 555 (22.6) | 361 (23.0) | 194 (21.9) |
| Hearing loss | 375 (15.3) | 231 (14.7) | 144 (16.3) |
| Heart disease | 339 (13.8) | 202 (12.9) | 137 (15.5) |
| Chronic obstructive pulmonary disease | 273 (11.1) | 176 (11.2) | 97 (11.0) |
| Memory problems | 253 (10.3) | 159 (10.1) | 94 (10.6) |
| Atrial Fibrillation | 193 (7.9) | 119 (7.6) | 74 (8.4) |
| Osteoporosis | 199 (8.1) | 131 (8.3) | 68 (7.7) |
| Obstructive sleep apnoea | 148 (6.0) | 94 (6.0) | 54 (6.1) |
| Stroke/ Mini stroke | 144 (5.9) | 91 (5.8) | 53 (6.0) |
| Sight loss | 117 (4.8) | 66 (4.2) | 51 (5.8) |
| Chronic kidney disease | 102 (4.2) | 63 (4.0) | 39 (4.4) |
| Liver problems | 89 (3.6) | 56 (3.6) | 33 (3.7) |
| Epilepsy | 53 (2.2) | 35 (2.2) | 18 (2.0) |
| Learning disability | 39 (1.6) | 25 (1.6) | 14 (1.6) |
| Dependence on alcohol or any substances | 30 (1.2) | 24 (1.5) | 6 (0.7) |

Shown are the number of subjects (%).

* Other conditions reported include thyroid, cancer, chronic fatigue, severe migraines, irritable bowel syndrome, Crohn’s disease, eczema, insomnia, bipolar, ADHD, Parkinson’s disease, bipolar, and Alzheimer’s.

# Table S3. Current first-choice health outcome priority by respondent sociodemographic factors and clusters of conditions

|  | **Maintaining independence** | **Keeping Alive** | **Reducing pain** | **Reducing other symptoms** | **P-value** |
| --- | --- | --- | --- | --- | --- |
| **All participants** (n=884) | 288 (32.6) | 386 (43.7) | 121 (13.7) | 89 (10.1) |  |
| **Age** |  |  |  |  |  |
| 45 - 65 y | 138 (28.6) | 229 (47.4) | 66 (13.7) | 50 (10.4) |  |
| ≥ 65 y | 149 (37.4) | 156 (39.2) | 54 (13.6) | 39 (9.8) | 0.034 |
| **Gender** |  |  |  |  |  |
| Female | 171 (34.8) | 201 (40.9) | 69 (14.1) | 50 (10.2) |  |
| Male | 114 (30.1) | 179 (47.2) | 47 (12.4) | 39 (10.3) | 0.278 |
| **Ethnicity** |  |  |  |  |  |
| White | 226 (32.4) | 361 (44.0) | 112 (13.6) | 82 (10.0) |  |
| Non-white | 13 (27.7) | 22 (46.8) | 6 (12.8) | 6 (13.8) | 0.860 |
| **Education** |  |  |  |  |  |
| None | 34 (24.1) | 62 (44.0) | 20 (14.2) | 25 (17.7) |  |
| GCSE, A Levels or equivalent | 136 (32.1) | 197 (46.5) | 56 (13.2) | 35 (8.3) |  |
| Higher education | 99 (41.4) | 98 (41.0) | 29 (12.1) | 13 (5.4) |  |
| Other (e.g., NVQ, nursing, missing) | 19 (23.8) | 29 (36.3) | 16 (20.0) | 16 (20.0) | <0.001 |
| **Employment status** |  |  |  |  |  |
| Working | 81 (27.3) | 160 (53.9) | 34 (11.5) | 22 (7.4) |  |
| Unemployed | 17 (24.6) | 22 (31.9) | 16 (23.2) | 14 (20.3) |  |
| Retired | 170 (38.2) | 173 (38.9) | 59 (13.3) | 43 (9.7) |  |
| Other (student, volunteer, missing) | 20 (27.4) | 31 (42.5) | 12 (16.4) | 10 (13.7) | <0.001 |
| **At high risk of COVID-19** |  |  |  |  |  |
| No | 70 (25.9) | 119 (44.1) | 48 (17.8) | 33 (12.2) |  |
| Yes | 214 (35.4) | 265 (43.8) | 73 (12.1) | 53 (8.8) | 0.008 |
| **Cardiometabolic conditions** |  |  |  |  |  |
| No | 84 (28.0) | 131 (43.7) | 43 (14.3) | 42 (14.0) |  |
| Yes | 204 (34.9) | 255 (43.7) | 78 (13.4) | 47 (8.1) | 0.018 |
| **Musculoskeletal or chronic pain** |  |  |  |  |  |
| No | 141 (34.9) | 184 (45.5) | 41 (10.2) | 38 (9.4) |  |
| Yes | 147 (30.6) | 202 (42.1) | 80 (16.7) | 51 (10.6) | 0.030 |
| **Mental health conditions** |  |  |  |  |  |
| No | 190 (33.9) | 255 (45.5) | 68 (12.1) | 47 (8.4) |  |
| Yes | 98 (30.3) | 131 (40.4) | 53 (16.4) | 42 (13.0) | 0.029 |
| **Respiratory conditions** |  |  |  |  |  |
| No | 196 (33.1) | 267 (45.0) | 77 (13.0) | 53 (8.9) |  |
| Yes | 92 (31.6) | 119 (40.9) | 44 (15.1) | 36 (12.4) | 0.281 |

Shown are the number of subjects (%) using row totals. P-values calculated by Chi squared test.

# Table S4. Association between patient’s first-choice health priority and sociodemographic factors and clusters of long-term conditions, unadjusted analyses

|  | **Odds ratio (95% CI)** | | | |
| --- | --- | --- | --- | --- |
|  | **Maintaining independence** | **Keeping Alive** | **Reducing pain** | **Reducing other symptoms** |
| **Age** |  |  |  |  |
| 45 - 65 y | Reference | Reference | Reference | Reference |
| ≥ 65 y | **1.49 (1.13, 1.99)** | **0.72 (0.55, 0.94)** | 0.99 (0.67, 1.46) | 0.94 (0.61, 1.46) |
| **Gender** |  |  |  |  |
| Female | Reference | Reference | Reference | Reference |
| Male | 0.81 (0.60, 1.07) | 1.29 (0.99, 1.69) | 0.87 (0.58, 1.29) | 1.01 (0.65, 1.57) |
| **Ethnicity** |  |  |  |  |
| White | Reference | Reference | Reference | Reference |
| Non-white | 0.80 (0.41, 1.54) | 1.12 (0.62, 2.02) | 0.93 (0.38, 2.23) | 1.32 (0.54, 3.20) |
| **Education** |  |  |  |  |
| None | Reference | Reference | Reference | Reference |
| GCSE, A Levels or equivalent | 1.49 (0.96, 2.30) | 1.11 (0.75, 1.62) | 0.92 (0.53, 1.60) | **0.42 (0.24, 0.73)** |
| Higher education | **2.23 (1.40, 3.54)** | 0.89 (0.58, 1.35) | 0.84 (0.45, 1.54) | **0.27 (0.13, 0.54)** |
| Other (e.g., NVQ, nursing, missing) | 0.98 (0.52, 1.87) | 0.72 (0.41, 1.27) | 1.51 (0.73, 3.12) | 1.16 (0.58, 2.33) |
| **Employment status** |  |  |  |  |
| Working | Reference | Reference | Reference | Reference |
| Unemployed | 0.87 (0.48, 1.60) | **0.40 (0.23, 0.70)** | **2.34 (1.20, 4.53)** | **3.18 (1.53, 6.60)** |
| Retired | **1.65 (1.20, 2.27)** | **0.54 (0.40, 0.73)** | 1.18 (0.75, 1.85) | 1.34 (0.78, 2.29) |
| Other (student, volunteer, missing) | 1.01 (0.57, 1.79) | **0.63 (0.38, 1.06)** | 1.52 (0.74, 3.11) | 1.98 (0.90, 4.40) |
| **At high risk of COVID-19** |  |  |  |  |
| No | Reference | Reference | Reference | Reference |
| Yes | **1.56 (1.14, 2.15)** | 0.99 (0.74, 1.32) | **0.63 (0.43, 0.94)** | 0.69 (0.44, 1.09) |
| **Cardiometabolic conditions** |  |  |  |  |
| No | Reference | Reference | Reference | Reference |
| Yes | **1.38 (1.02, 1.87)** | 1.00 (0.76, 1.32) | 0.92 (0.62, 1.38) | **0.54 (0.35, 0.84)** |
| **Musculoskeletal or chronic pain** |  |  |  |  |
| No | Reference | Reference | Reference | Reference |
| Yes | 0.82 (0.62, 1.09) | 0.87 (0.67, 1.13) | **1.77 (1.18, 2.65)** | 1.15 (0.74, 1.78) |
| **Mental health conditions** |  |  |  |  |
| No | Reference | Reference | Reference | Reference |
| Yes | 0.84 (0.63, 1.13) | 0.81 (0.62, 1.07) | 1.42 (0.96, 2.09) | **1.63 (1.05, 2.53)** |
| **Respiratory conditions** |  |  |  |  |
| No | Reference | Reference | Reference | Reference |
| Yes | 0.94 (0.69, 1.26) | 0.84 (0.64, 1.12) | 1.19 (0.80, 1.78) | 1.44 (0.92, 2.25) |

Unadjusted models. CI=confidence interval.

Odds ratio <1 indicates low health priority, whereas odds ratio >1 indicate high health priority.

Bold indicates statistical significance, P<0.05.

# Table S5. Association between patient’s first-choice health priority and sociodemographic factors and clusters of long-term conditions, adjusted for age and gender

|  | **Odds ratio (95% CI)** | | | |
| --- | --- | --- | --- | --- |
|  | **Maintaining independence** | **Keeping alive** | **Reducing pain** | **Reducing other symptoms** |
| **Age** |  |  |  |  |
| 45 - 65 y | Reference | Reference | Reference | Reference |
| ≥ 65 y | **1.55 (1.16, 2.06)** | **0.70 (0.53, 0.92)** | 0.96 (0.65, 1.43) | 0.94 (0.61, 1.47) |
| **Gender** |  |  |  |  |
| Female | Reference | Reference | Reference | Reference |
| Male | 0.75 (0.56, 1.00) | **1.37 (1.04, 1.80)** | 0.86 (0.58, 1.29) | 1.04 (0.67, 1.63) |
| **Ethnicity** |  |  |  |  |
| White | Reference | Reference | Reference | Reference |
| Non-white | 1.00 (0.51, 1.96) | 0.93 (0.51, 1.70) | 0.97 (0.40, 2.38) | 1.19 (0.48, 2.93) |
| **Education** |  |  |  |  |
| None | Reference | Reference | Reference | Reference |
| GCSE, A Levels or equivalent | **1.73 (1.11, 2.71)** | 1.02 (0.69, 1.51) | 0.91 (0.52, 1.59) | **0.38 (0.22, 0.67)** |
| Higher education | **2.57 (1.60, 4.14)** | 0.81 (0.53, 1.25) | 0.83 (0.44, 1.53) | **0.25 (0.12, 0.52)** |
| Other (e.g., NVQ, nursing, missing) | 0.93 (0.48, 1.81) | 0.77 (0.43, 1.37) | 1.30 (0.60, 2.79) | 1.37 (0.67, 2.78) |
| **Employment status** |  |  |  |  |
| Working | Reference | Reference | Reference | Reference |
| Unemployed | 0.88 (0.48, 1.61) | **0.40 (0.23, 0.69)** | **2.41 (1.23, 4.69)** | **3.07 (1.48, 6.38)** |
| Retired | 1.17 (0.75, 1.82) | 0.73 (0.48, 1.10) | 1.02 (0.55, 1.89) | 1.72 (0.83, 3.53) |
| Other (student, volunteer, missing) | 0.95 (0.52, 1.73) | 0.66 (0.39, 1.14) | 1.33 (0.62, 2.88) | 2.28 (1.02, 5.11) |
| **At high risk of COVID-19** |  |  |  |  |
| No | Reference | Reference | Reference | Reference |
| Yes | **1.60 (1.15, 2.21)** | 0.98 (0.73, 1.31) | **0.64 (0.42, 0.95)** | 0.69 (0.43, 1.09) |
| **Cardiometabolic conditions** |  |  |  |  |
| No | Reference | Reference | Reference | Reference |
| Yes | 1.32 (0.96, 1.82) | 1.09 (0.81, 1.47) | 0.86 (0.56, 1.32) | **0.53 (0.33, 0.84)** |
| **Musculoskeletal or chronic pain** |  |  |  |  |
| No | Reference | Reference | Reference | Reference |
| Yes | **0.74 (0.55, 1.00)** | 0.97 (0.74, 1.28) | **1.65 (1.09, 2.50)** | 1.20 (0.76, 1.89) |
| **Mental health conditions** |  |  |  |  |
| No | Reference | Reference | Reference | Reference |
| Yes | 0.96 (0.70, 1.32) | **0.71 (0.52, 0.95)** | 1.47 (0.97, 2.23) | **1.62 (1.02, 2.57)** |
| **Respiratory conditions** |  |  |  |  |
| No | Reference | Reference | Reference | Reference |
| Yes | 1.01 (0.74, 1.37) | 0.81 (0.60, 1.08) | 1.15 (0.76, 1.74) | 1.43 (0.91, 2.25) |

Models are adjusted by age (continuous) and gender (female or male). CI=confidence interval.

Odds ratio <1 indicates low health priority, whereas odds ratio >1 indicate high health priority.

Bold indicates statistical significance, P<0.05.

# Table S6. Association between patient’s first-choice health priority and sociodemographic factors and clusters of long-term conditions, fully adjusted

|  | **Odds ratio (95% CI)** | | | |
| --- | --- | --- | --- | --- |
|  | **Maintaining independence** | **Keeping alive** | **Reducing pain** | **Reducing other symptoms** |
| **Age** |  |  |  |  |
| 45 - 65 y | Reference | Reference | Reference | Reference |
| ≥ 65 y | 1.36 (0.86, 2.15) | 0.77 (0.50, 1.18) | 1.13 (0.59, 2.15) | 0.84 (0.39, 1.79) |
| **Gender** |  |  |  |  |
| Female | Reference | Reference | Reference | Reference |
| Male | **0.68 (0.49, 0.93)** | 1.27 (0.95, 1.71) | 0.98 (0.64, 1.50) | 1.34 (0.82, 2.19) |
| **Ethnicity** |  |  |  |  |
| White | Reference | Reference | Reference | Reference |
| Non-white | 0.90 (0.45, 1.79) | 1.03 (0.55, 1.91) | 0.97 (0.39, 2.41) | 1.33 (0.49, 3.64) |
| **Education** |  |  |  |  |
| None | Reference | Reference | Reference | Reference |
| GCSE, A Levels or equivalent | **1.65 (1.04, 2.62)** | 0.95 (0.64, 1.42) | 1.01 (0.57, 1.79) | **0.43 (0.24, 0.78)** |
| Higher education | **2.64 (1.50, 4.04)** | 0.73 (0.47, 1.14) | 0.98 (0.51, 1.86) | **0.29 (0.14, 0.61)** |
| Other (e.g., NVQ, nursing, missing) | 0.86 (0.42, 1.76) | 0.77 (0.42, 1.42) | 1.37 (0.62, 3.07) | 1.44 (0.67, 3.10) |
| **Employment status** |  |  |  |  |
| Working | Reference | Reference | Reference | Reference |
| Unemployed | 1.14 (0.60, 2.17) | **0.42 (0.23, 0.75)** | 1.88 (0.93, 3.82) | **2.17 (0.98, 4.78)** |
| Retired | 1.34 (0.83, 2.15) | 0.65 (0.42, 1.02) | 1.07 (0.54, 2.11) | 1.66 (0.74, 3.74) |
| Other (student, volunteer, missing) | 0.96 (0.49, 1.76) | 0.75 (0.42, 1.33) | 1.13 (0.49, 2.57) | 1.97 (0.81, 4.79) |
| **At high risk of COVID-19** |  |  |  |  |
| No | Reference | Reference | Reference | Reference |
| Yes | **1.52 (1.07, 2.18)** | 0.91 (0.66, 1.25) | 0.67 (0.43, 1.05) | 0.90 (0.53, 1.51) |
| **Cardiometabolic conditions** |  |  |  |  |
| No | Reference | Reference | Reference | Reference |
| Yes | 1.48 (1.05, 2.08) | 1.04 (0.76, 1.41) | 0.84 (0.54, 1.30) | **0.46 (0.28, 0.77)** |
| **Musculoskeletal or chronic pain** |  |  |  |  |
| No | Reference | Reference | Reference | Reference |
| Yes | 0.79 (0.58, 1.08) | 1.00 (0.75, 1.33) | 1.53 (0.99, 2.35) | 1.02 (0.63, 1.66) |
| **Mental health conditions** |  |  |  |  |
| No | Reference | Reference | Reference | Reference |
| Yes | 0.92 (0.66, 1.29) | 0.81 (0.59, 1.10) | 1.34 (0.87, 2.10) | 1.45 (0.87, 2.42) |
| **Respiratory conditions** |  |  |  |  |
| No | Reference | Reference | Reference | Reference |
| Yes | 1.25 (0.89, 1.76) | 0.86 (0.63, 1.17) | 0.87 (0.56, 1.37) | 1.07 (0.64, 1.79) |

Models are fully adjusted. CI=confidence interval.

Odds ratio <1 indicates low health priority, whereas odds ratio >1 indicate high health priority.

Bold indicates statistical significance, P<0.05.

# Table S7. Before COVID-19 first-choice health outcome priority by respondent characteristics, N (%) row totals

|  | **Maintaining independence** | **Keeping alive** | **Reducing pain** | **Reducing other symptoms** | **P-value** |
| --- | --- | --- | --- | --- | --- |
| **All participants** (n=884) | 301 (34.1) | 335 (37.9) | 156 (17.7) | 92 (10.4) |  |
| **Age** |  |  |  |  |  |
| 45 - 65 y | 141 (29.2) | 198 (41.0) | 88 (18.2) | 56 (11.6) |  |
| ≥ 65 y | 160 (40.2) | 136 (34.2) | 67 (16.8) | 35 (8.8) | 0.006 |
| **Gender** |  |  |  |  |  |
| Female | 181 (36.9) | 162 (33.0) | 96 (19.6) | 52 (10.6) |  |
| Male | 118 (31.1) | 168 (44.3) | 55 (14.5) | 38 (10.0) | 0.006 |
| **Ethnicity** |  |  |  |  |  |
| White | 279 (34.0) | 315 (38.4) | 142 (17.3) | 85 (10.4) |  |
| Non-white | 14 (29.8) | 19 (40.4) | 9 (19.2) | 5 (10.6) | 0.946 |
| **Education** |  |  |  |  |  |
| None | 43 (30.5) | 45 (31.9) | 27 (19.2) | 26 (18.4) |  |
| GCSE, A Levels or equivalent | 134 (31.6) | 174 (41.0) | 79 (18.6) | 37 (8.7) |  |
| Higher education | 98 (41.0) | 87 (36.4) | 36 (15.1) | 18 (7.5) |  |
| Other (e.g., NVQ, nursing, missing) | 26 (32.5) | 29 (36.3) | 14 (17.5) | 11 (13.8) | 0.011 |
| **Employment status** |  |  |  |  |  |
| Working | 89 (30.0) | 134 (45.1) | 48 (16.2) | 26 (8.8) |  |
| Unemployed | 18 (26.1) | 19 (27.5) | 14 (20.3) | 18 (26.1) |  |
| Retired | 176 (39.6) | 159 (35.7) | 73 (16.4) | 37 (8.3) |  |
| Other (student, volunteer, missing) | 18 (24.7) | 23 (31.5) | 21 (28.8) | 11 (15.1) | <0.001 |
| **At high risk of COVID-19** |  |  |  |  |  |
| No | 80 (29.6) | 102 (37.8) | 52 (19.3) | 36 (13.3) |  |
| Yes | 219 (36.2) | 231 (38.2) | 99 (16.4) | 56 (9.3) | 0.101 |
| **Cardiometabolic conditions** |  |  |  |  |  |
| No | 90 (30.0) | 102 (34.0) | 69 (23.0) | 39 (13.0) |  |
| Yes | 211 (36.1) | 233 (39.9) | 87 (14.9) | 53 (9.1) | 0.003 |
| **Musculoskeletal or chronic pain** |  |  |  |  |  |
| No | 141 (34.9) | 158 (39.1) | 62 (15.4) | 43 (10.6) |  |
| Yes | 160 (33.3) | 177 (36.9) | 94 (19.6) | 49 (10.2) | 0.437 |
| **Mental health conditions** |  |  |  |  |  |
| No | 205 (36.6) | 220 (39.3) | 92 (16.4) | 43 (7.7) |  |
| Yes | 96 (29.6) | 115 (35.5) | 64 (19.8) | 49 (15.1) | 0.001 |
| **Respiratory conditions** |  |  |  |  |  |
| No | 198 (33.4) | 227 (38.3) | 108 (18.2) | 60 (10.1) |  |
| Yes | 103 (35.4) | 108 (37.1) | 48 (16.5) | 32 (11.0) | 0.856 |

Shown are the number of subjects (%) using row totals. P-values calculated by Chi squared test.

# Figure S2. Health outcome priority score before COVID-19 and current


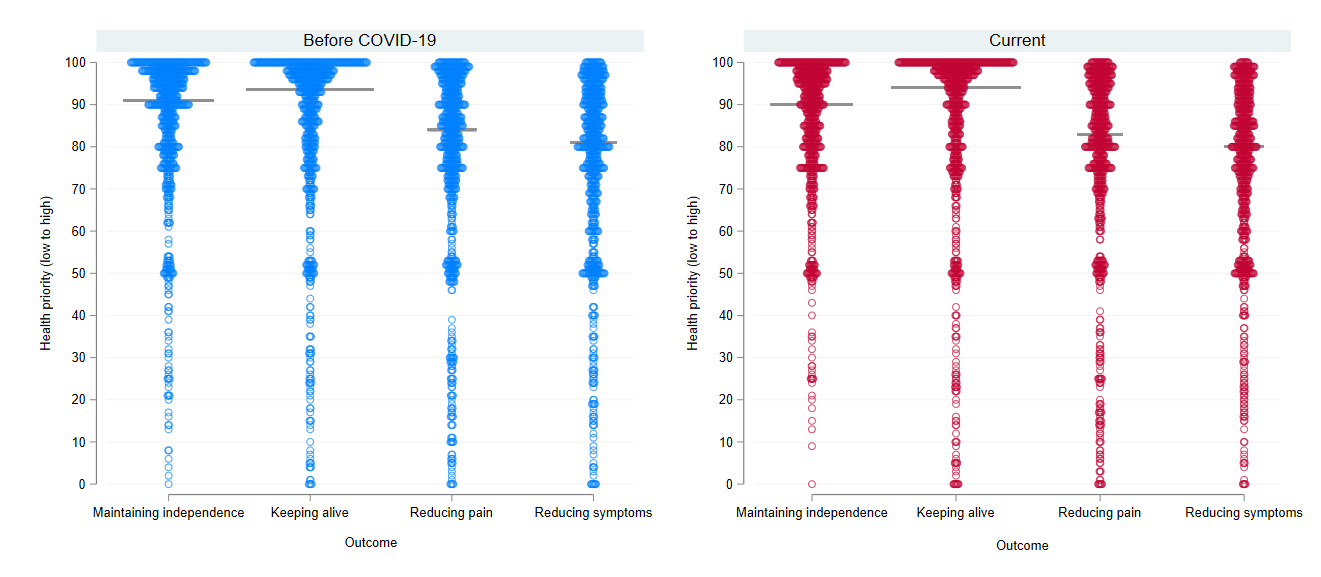


Reference line represents the median value of health prioritisation on the scale of 0 to 100.

Median (interquartile range):

Before COVID-19: maintaining independence 91 [79 - 97.5], keeping alive 93.5 [79.5 - 98], reducing pain 84 [72 - 94], reducing other symptoms 81 [65 - 92].

Current: maintaining independence 90 [79 - 97], keeping alive 94 [79 - 99], reducing pain 83 [72 - 94], reducing other symptoms 80 [64 - 90].

# Figure S3. Comparing the health outcome priority score before COVID-19 and current

# Table S8. The health outcome prioritisation tool was easy to complete by patient’s sociodemographic factors and clusters of long-term conditions, N (%)

|  | **Strongly agree** | **Agree** | **Neither agree nor disagree** | **Disagree** | **Strongly disagree** | **P-value** |
| --- | --- | --- | --- | --- | --- | --- |
| **Age** |  |  |  |  |  |  |
| 45 - 65 y | 99 (20.5) | 211 (43.7) | 94 (19.5) | 46 (9.5) | 33 (6.8) |  |
| ≥ 65 y | 63 (15.8) | 198 (49.8) | 90 (22.6) | 30 (7.5) | 17 (4.3) | 0.064 |
| **Gender** |  |  |  |  |  |  |
| Female | 87 (17.7) | 230 (46.8) | 97 (19.8) | 47 (9.6) | 30 (6.1) |  |
| Male | 73 (19.3) | 174 (45.9) | 85 (22.4) | 28 (7.4) | 19 (5.0) | 0.611 |
| **Ethnicity** |  |  |  |  |  |  |
| White | 154 (18.8) | 380 (46.3) | 165 (20.1) | 75 (9.1) | 47 (5.7) |  |
| Non-white | 6 (12.8) | 21 (44.7) | 17 (36.2) | 0 (0.0) | 3 (6.4) | 0.030 |
| **Education** |  |  |  |  |  |  |
| None | 19 (13.5) | 68 (48.2) | 46 (32.6) | 6 (4.3) | 2 (1.4) |  |
| GCSE, A Levels or equivalent | 84 (19.8) | 207 (48.8) | 74 (17.5) | 35 (8.3) | 24 (5.7) |  |
| Higher education | 45 (18.8) | 102 (42.7) | 43 (18.0) | 30 (12.6) | 19 (8.0) |  |
| Other (e.g., NVQ, nursing, missing) | 15 (18.8) | 34 (42.5) | 21 (26.3) | 5 (6.3) | 5 (6.3) | 0.001 |
| **Employment status** |  |  |  |  |  |  |
| Working | 63 (21.2) | 137 (46.1) | 51 (17.2) | 27 (9.1) | 19 (6.4) |  |
| Unemployed | 12 (17.4) | 30 (43.5) | 18 (26.1) | 7 (10.1) | 2 (2.9) |  |
| Retired | 72 (16.2) | 213 (47.9) | 101 (22.7) | 36 (8.1) | 23 (5.2) |  |
| Other (student, volunteer, missing) | 16 (21.9) | 31 (42.5) | 14 (19.2) | 6 (8.2) | 6 (8.2) | 0.624 |
| **At high risk of COVID-19** |  |  |  |  |  |  |
| No | 50 (18.5) | 135 (50.0) | 55 (20.4) | 14 (5.2) | 16 (5.9) |  |
| Yes | 111 (18.4) | 273 (45.1) | 126 (20.8) | 62 (10.3) | 33 (5.5) | 0.161 |
| **Cardiometabolic conditions** |  |  |  |  |  |  |
| No | 51 (17.0) | 130 (43.3) | 61 (20.3) | 33 (11.0) | 25 (8.3) |  |
| Yes | 112 (19.2) | 281 (48.1) | 123 (21.1) | 43 (7.4) | 25 (4.3) | 0.035 |
| **Musculoskeletal or chronic pain** |  |  |  |  |  |  |
| No | 79 (19.6) | 174 (43.1) | 93 (23.0) | 31 (7.7) | 27 (6.7) |  |
| Yes | 84 (17.5) | 237 (49.4) | 91 (19.0) | 45 (9.4) | 23 (4.8) | 0.182 |
| **Mental health conditions** |  |  |  |  |  |  |
| No | 93 (16.6) | 266 (47.5) | 113 (20.2) | 54 (9.6) | 34 (6.1) |  |
| Yes | 70 (21.6) | 145 (44.8) | 71 (21.9) | 22 (6.8) | 16 (4.9) | 0.213 |
| **Respiratory conditions** |  |  |  |  |  |  |
| No | 123 (20.7) | 268 (45.2) | 118 (19.9) | 54 (9.1) | 30 (5.1) |  |
| Yes | 40 (13.8) | 143 (49.1) | 66 (22.7) | 22 (7.6) | 20 (6.9) | 0.083 |

Shown are the number of subjects (%) using row totals. P-values calculated by Chi squared test.

# Table S9. The health outcome prioritisation tool was relevant to my healthcare by patient’s sociodemographic factors and clusters of long-term conditions, N (%)

|  | **Strongly agree** | **Agree** | **Neither agree nor disagree** | **Disagree** | **Strongly disagree** | **P-value** |
| --- | --- | --- | --- | --- | --- | --- |
| **Age** |  |  |  |  |  |  |
| 45 - 65 y | 38 (7.8) | 224 (46.4) | 151 (31.3) | 49 (10.1) | 21 (4.4) |  |
| ≥ 65 y | 36 (9.1) | 189 (47.7) | 134 (33.8) | 23 (5.8) | 14 (3.5) | 0.180 |
| **Gender** |  |  |  |  |  |  |
| Female | 36 (7.4) | 229 (46.8) | 160 (32.7) | 39 (8.0) | 25 (5.1) |  |
| Male | 37 (9.8) | 176 (46.4) | 124 (32.7) | 32 (8.4) | 10 (2.6) | 0.312 |
| **Ethnicity** |  |  |  |  |  |  |
| White | 70 (8.6) | 376 (45.9) | 270 (33.0) | 70 (8.6) | 33 (4.0) |  |
| Non-white | 3 (6.4) | 29 (61.7) | 12 (25.5) | 1 (2.1) | 2 (4.3) | 0.230 |
| **Education** |  |  |  |  |  |  |
| None | 12 (8.5) | 75 (53.2) | 44 (31.2) | 7 (5.0) | 3 (2.1) |  |
| GCSE, A Levels or equivalent | 33 (7.8) | 207 (48.9) | 141 (33.3) | 27 (6.4) | 15 (3.6) |  |
| Higher education | 21 (8.8) | 91 (38.1) | 81 (33.9) | 34 (14.2) | 12 (5.0) |  |
| Other (e.g., NVQ, nursing, missing) | 10 (12.7) | 41 (51.9) | 19 (24.1) | 4 (5.1) | 5 (6.3) | 0.005 |
| **Employment status** |  |  |  |  |  |  |
| Working | 23 (7.8) | 129 (43.6) | 102 (34.5) | 27 (9.1) | 15 (5.1) |  |
| Unemployed | 8 (11.6) | 35 (50.7) | 19 (27.5) | 7 (10.1) | 0 (0.0) |  |
| Retired | 39 (8.8) | 214 (48.2) | 139 (31.3) | 34 (7.7) | 18 (4.1) |  |
| Other (student, volunteer, missing) | 6 (8.2) | 36 (49.3) | 25 (34.3) | 4 (5.5) | 2 (2.7) | 0.733 |
| **At high risk of COVID-19** |  |  |  |  |  |  |
| No | 27 (10.0) | 149 (55.4) | 66 (24.5) | 14 (5.2) | 13 (4.8) |  |
| Yes | 47 (7.8) | 263 (43.5) | 217 (35.9) | 56 (9.3) | 21 (3.5) | 0.001 |
| **Cardiometabolic conditions** |  |  |  |  |  |  |
| No | 25 (8.3) | 118 (39.3) | 104 (34.7) | 34 (11.3) | 19 (6.3) |  |
| Yes | 51 (8.8) | 296 (50.9) | 181 (31.1) | 38 (6.5) | 16 (2.8) | 0.001 |
| **Musculoskeletal or chronic pain** |  |  |  |  |  |  |
| No | 26 (6.5) | 180 (44.7) | 138 (34.2) | 40 (9.9) | 19 (4.7) |  |
| Yes | 50 (10.4) | 234 (48.9) | 147 (30.7) | 32 (6.7) | 16 (3.3) | 0.048 |
| **Mental health conditions** |  |  |  |  |  |  |
| No | 43 (7.7) | 257 (46.1) | 182 (32.6) | 51 (9.1) | 25 (4.5) |  |
| Yes | 33 (10.2) | 157 (48.5) | 103 (31.8) | 21 (6.5) | 10 (3.1) | 0.338 |
| **Respiratory conditions** |  |  |  |  |  |  |
| No | 52 (8.8) | 260 (43.9) | 204 (34.5) | 51 (8.36) | 25 (4.2) |  |
| Yes | 24 (8.3) | 154 (53.1) | 81 (27.9) | 21 (7.2) | 10 (3.5) | 0.143 |

Shown are the number of subjects (%) using row totals. P-values calculated by Chi squared test.

# Table S10. The health outcome prioritisation tool will be useful in communicating what my priorities are to my doctor by patient’s sociodemographic factors and clusters of long-term conditions, N (%)

|  | **Strongly agree** | **Agree** | **Neither agree nor disagree** | **Disagree** | **Strongly disagree** | **P-value** |
| --- | --- | --- | --- | --- | --- | --- |
| **Age** |  |  |  |  |  |  |
| 45 - 65 y | 41 (8.5) | 238 (49.3) | 127 (26.3) | 48 (9.9) | 29 (6.0) |  |
| ≥ 65 y | 44 (11.1) | 201 (50.5) | 110 (27.6) | 29 (7.3) | 14 (3.5) | 0.183 |
| **Gender** |  |  |  |  |  |  |
| Female | 50 (10.2) | 243 (49.5) | 123 (25.1) | 52 (10.6) | 23 (4.7) |  |
| Male | 34 (9.0) | 190 (50.1) | 112 (29.6) | 23 (6.1) | 20 (5.3) | 0.127 |
| **Ethnicity** |  |  |  |  |  |  |
| White | 77 (9.4) | 407 (49.6) | 224 (27.3) | 74 (9.0) | 39 (4.8) |  |
| Non-white | 7 (14.9) | 26 (55.3) | 11 (23.4) | 1 (2.1) | 2 (4.3) | 0.354 |
| **Education** |  |  |  |  |  |  |
| None | 16 (11.4) | 82 (58.2) | 27 (19.2) | 10 (7.1) | 6 (4.3) |  |
| GCSE, A Levels or equivalent | 46 (10.9) | 215 (50.7) | 115 (27.1) | 29 (6.8) | 19 (4.5) |  |
| Higher education | 14 (5.9) | 104 (43.5) | 74 (31.0) | 32 (13.4) | 15 (6.3) |  |
| Other (e.g., NVQ, nursing, missing) | 10 (12.5) | 39 (48.8) | 22 (27.5) | 6 (7.5) | 3 (3.8) | 0.024 |
| **Employment status** |  |  |  |  |  |  |
| Working | 20 (6.7) | 151 (50.8) | 84 (28.3) | 28 (9.4) | 14 (4.7) |  |
| Unemployed | 9 (13.0) | 37 (53.6) | 11 (15.9) | 7 (10.1) | 5 (7.3) |  |
| Retired | 48 (10.8) | 217 (48.8) | 123 (27.6) | 38 (8.5) | 19 (4.3) |  |
| Other (student, volunteer, missing) | 9 (12.3) | 35 (48.0) | 20 (27.4) | 4 (5.5) | 5 (6.9) | 0.504 |
| **At high risk of COVID-19** |  |  |  |  |  |  |
| No | 33 (12.2) | 146 (54.1) | 58 (21.5) | 19 (7.0) | 14 (5.2) |  |
| Yes | 51 (8.4) | 292 (48.3) | 177 (29.3) | 56 (9.3) | 29 (4.8) | 0.052 |
| **Cardiometabolic conditions** |  |  |  |  |  |  |
| No | 28 (9.3) | 142 (47.3) | 74 (24.7) | 32 (10.7) | 24 (8.0) |  |
| Yes | 58 (9.9) | 298 (51.0) | 164 (28.1) | 45 (7.7) | 19 (3.3) | 0.013 |
| **Musculoskeletal or chronic pain** |  |  |  |  |  |  |
| No | 28 (6.9) | 192 (47.5) | 119 (29.5) | 43 (10.6) | 22 (5.5) |  |
| Yes | 58 (12.1) | 248 (51.7) | 119 (24.8) | 34 (7.1) | 21 (4.4) | 0.016 |
| **Mental health conditions** |  |  |  |  |  |  |
| No | 49 (8.8) | 273 (48.8) | 164 (29.3) | 45 (8.0) | 29 (5.2) |  |
| Yes | 37 (11.4) | 167 (51.5) | 74 (22.8) | 32 (9.9) | 14 (4.3) | 0.192 |
| **Respiratory conditions** |  |  |  |  |  |  |
| No | 62 (10.5) | 285 (48.1) | 167 (28.2) | 51 (8.6) | 28 (4.7) |  |
| Yes | 24 (8.3) | 155 (53.3) | 71 (24.4) | 26 (8.9) | 15 (5.2) | 0.530 |

Shown are the number of subjects (%) using row totals. P-values calculated by Chi squared test.
